# Research on the Influence of Tolerance of Opportunistic Behaviors of Channel Boundaries on the Choice of Response Strategies

**DOI:** 10.3389/fpsyg.2022.823416

**Published:** 2022-04-08

**Authors:** Jinsong Chen, Zhaoxia Liu, Ruoqian Hu

**Affiliations:** Department of Marketing, College of Business Administration, Guizhou University of Finance and Economics, Guiyang, China

**Keywords:** opportunistic behavior, opportunistic behavior tolerance, opportunistic behavior response strategy selection, contract formulation, psychological contract

## Abstract

In the Chinese society, border agents in channel transactions will choose different opportunistic behavior response strategies to the tolerance of other members based on the relationship between the two parties. Based on 206 valid questionnaires collected, structural equation model and regression analysis were used to investigate the influence of opportunistic behavior tolerance on response strategy selection. The results show that the channel boundary personnel's tolerance to opportunistic behavior (based on work or personal) negatively influences their choice of a positive response strategy and positively influences their choice of a negative response strategy. Among the mediating effects of contract formulation, transaction terms have a positive effect on the choice of negative response strategies based on the work and individual opportunistic behavior tolerance and have no mediating effect on the choice of positive response strategies; the contingency clause has no mediating effect on the choice of positive response strategies based on individual opportunistic behavior tolerance.

## Introduction

With an increase in channel modes, opportunistic behaviors between channels become more frequent. Opportunistic behavior is an act of “seek self-interest by trickery” that refers to a channel member at the expense of the other channel member's interests to maximize benefits, including fraud, breach of contract, dishonesty, and distortion of facts (Williamson, 1975). Channel transactions involve business contacts between both sides and include interpersonal communication. The tolerance of opportunistic behavior based on personal and work will affect the completeness of the transaction terms and the comprehensiveness of the contingency-handling terms of both parties, and they will adopt different response strategies to the opportunistic behavior of the other party.

The following issues are worth exploring: with an increase in the number of transactions, the accumulation of feelings of channel boundary personnel, and the establishment of a specific transaction basis, channel boundary personnel will take opportunistic behavior to maximize their interests. When the other party implements opportunistic behavior, what response strategy should they choose? How to make more perfect and detailed contract terms for different suppliers to reduce the risk of cooperation? Based on this, from the perspective of channel boundary personnel's tolerance of opportunistic behavior, this article studies which response strategies channel boundary personnel choose based on different roles' tolerance of opportunistic behavior and explores the mediating effect of contract formulation. First, it is hoped that this study can reveal the relationship between opportunistic tolerance, contract formulation, and response strategy selection of channel boundary personnel and enrich the research on contract formulation and response strategy selection of opportunistic behavior. Second, it can expand the application of contract formulation in the governance of opportunistic behavior, to provide guidance for channel boundary personnel to choose opportunistic behavior response strategies and to improve the level of contract formulation for both organizations and managers, in order to maintain a healthy channel relationship.

## Literature Review

### Research on Tolerance of Opportunistic Behavior

Opportunistic behavior and opportunistic behavior tolerance are two different concepts involving different aspects of the parties. Opportunistic behavior is an essential hypothesis in transaction cost theory, which describes the motivation of a channel provider. One party use trick, deception, and concealment of information to seek its interests (Williamson, 1975). In contrast, opportunistic behavior tolerance refers to one party's willingness to tolerate or forgive the other party's opportunistic behavior, which measures one party's tolerance to the other party's implementation of opportunistic behavior (Qian and Zhang, 2018). In the channel transaction, channel members' channeling goods, breach of contract, rip-off behavior is typical, bringing specific negative influences to the channel relationship. However, as far as the actual channel management is concerned, the tolerance of some opportunistic behaviors also exists objectively (Wathne and Heide, 2000). The moderating orientation of the enterprise owner will affect the limited degree of its tolerance to the opportunistic behavior of the other party. Because the enterprises that prevent orientation have a lower tolerance to the risk of loss, the threshold of the tolerance to opportunistic behavior of the enterprise owner is lower than the enterprises that promote orientation (Qian and Zhang, 2018).

Furthermore, as proprietary assets increase, one party will compare transaction costs to actual losses and tend to tolerate and learn from weak forms of opportunistic behavior by its partner (Luo et al., 2015). In addition, when the power and dependence of channel members are not equal, the party with solid dependence and the party with small channel power will tolerate the opportunistic behavior of the other party (Chen et al., 2019). Based on Chen's (2017) research, this article argues that individual-based opportunistic behavior tolerance is an attitude toward the opportunistic behavior of channel cooperation organizations based on the personal relationship between border personnel. Work-based opportunistic behavior tolerance is an attitude toward the opportunistic behavior of channel cooperation organizations based on the responsibilities of border personnel in the organization.

### Related Research on Opportunistic Behavior Response Strategies

Hirschman (1970) was the first to propose response strategies, including exit, voice, and loyalty, known as the EVL model, which is mainly applied in enterprise management. Based on this, Rusbult et al. (1982) added neglect, which means that he does not react to the other party's behavior, and will slowly alienate the other party and modify the model to the EVLN model to study the general reaction to the unsatisfactory situation in the exchange relationship. The EVLN model has been applied to various organizational environments, including employee attitudes toward colleagues, psychological contract, role conflict, and autonomy. The application of the response strategy in channel research started with Ping (1993), who mainly studied what response strategy channel members would adopt when the channel relationship was destroyed. Based on the EVLN model, Seggie et al. (2013) subdivided the EVLN model into six levels in the study of channel members' tolerance to opportunistic behavior, including passive acceptance, constructive discussion, complaint, alienation, threatening exit, and sign out. The six opportunistic response mechanisms can be divided into positive and negative ones. The positive ones include positive suggestions and passive acceptance, while the negative ones include expressing complaints, alienating relationships, threatening exit, and signing out. Scholars have explored the antecedents of opportunistic behavior response strategies and confirmed that enterprise regulation orientation (Qian and Zhang, 2018), psychological contract violation (Kingshott et al., 2020), channel power (Zhang et al., 2016), and human relationship (Zhang and Yin, 2019) would affect channel members' choice of neglect, appeal, exit, and loyalty. Based on the Seggie et al.'s classification of response strategies to opportunistic behaviors, this study explores the influence of tolerance of opportunistic behaviors on the choice of response strategies to opportunistic behaviors.

### Research on Contract Formulation

With the continuous expansion of market scale and the increase in channel mode, contract plays an increasingly important role in transactions. Enterprises usually sign contracts to clarify transaction items and improve management efficiency (Wang et al., 2019). Based on transaction cost theory, many researchers have explored the role of contracts in managing interorganizational relations (Zhang et al., 2017; Yang et al., 2018). Song and Chen (2020) pointed out that suppliers and distributors often use contracts to protect their obligations, responsibilities, and roles in the transaction cooperation, reduce the risks and uncertainties in the transaction relationship, and reduce the possibility of opportunistic behavior. Some scholars also believe that the contract itself is highly binding, and signing the contract means distrust of the other party (Ghoshal and Moran, 1996), which may increase the generation of retaliation and resistance (Kashyap and Murtha, 2017), leading to the failure to establish a good relationship between channel members and promote the occurrence of opportunistic behaviors. The above views have been verified in empirical studies, but the research on contracts is too single. Luo (2002) divides contract clauses into terms specificity and contingency adaptability. The precise terms of the transaction relate to the degree of detail that is generally foreseeable about the relevant matters; a comprehensive contingency clause refers to whether the contingency has been dealt with in detail. Zhou et al. (2013) studied the inhibiting effect of detailed transaction clauses and contingency-handling clauses on opportunistic behaviors. They verified the influence of the long-term orientation of enterprises and contract clauses on opportunistic behaviors. This study draws on Luo's division of contract formulation and explores its mediating role in the influence of channel boundary personnel's opportunistic behavior tolerance on response strategy choice.

Through the review of relevant literature, it is found that, first, in the case of cooperation between the two parties, channel boundary personnel will choose different response strategies to the opportunistic behavior of the other party based on their different roles, but the existing research on this aspect is insufficient. Second, the opportunistic behavior response strategy mainly focuses on passive acceptance, constructive discussion, and withdrawal, and the research on the choice of an opportunistic behavior response strategy is not enough. Third, the study of contract making in opportunistic behavior tolerance is insufficient, and it is only studied as a governance mechanism. However, it plays a more critical role in tolerating opportunistic behavior. Therefore, from the perspective of channel boundary personnel's tolerance of opportunistic behaviors, this article explores how channel boundary personnel choose response strategies of opportunistic behaviors based on different roles' tolerance of opportunistic behaviors and explores the mediating effect of contract formulation.

## Materials and Methods

### Transaction Cost Theory

Coase (1937) first proposed the theory of transaction cost, which was applied in economics. He believed that the phenomenon of using price mechanisms in the transaction is universal, and the cost generated in this process is the transaction cost. Williamson (1975) further explored the theory of transaction costs and pointed out that the costs were generated from transactions, including asset specificity, transaction uncertainty, and transaction frequency. Asset specificity is the dedicated investment of a portion of an asset for a specific activity. The degree of asset specificity is positively proportional to the probability of opportunistic behaviors. The more invested assets, the more likely they are to encounter opportunistic behaviors of channel members of the opposite party. Transaction uncertainty refers to the environmental and risk uncertainty that may exist in the process of channel transaction, which will increase the probability of opportunistic behavior. Transaction frequency can effectively reduce transaction costs. With the increase in transaction frequency, both parties will have more and more input costs and higher exit barriers. From the perspective of profit maximization, both channel members will pay more attention to long-term profits and reduce the frequency of opportunism to the other party. The two hypotheses of transaction cost are bounded rationality and opportunism, respectively. First, both parties are bounded rational in the transaction process, so it is impossible to predict all future situations in place, and the contract signed by both parties cannot contain all situations. When unexpected situations occur, both parties will increase the transaction costs in the process of bargaining. The second is opportunistic behavior. Both parties will act opportunistic behavior to maximize their interests in the transaction process. To avoid the occurrence of opportunistic behavior, both sides of the transaction need to establish a contract to restrain each other's behavior, which will increase the transaction cost (Williamson, 1975, 1985). Based on the scholars' research, this study uses the uncertainty and opportunistic hypothesis in transaction cost theory to explore the influence of opportunistic behavior tolerance on the choice of response strategy and the mediating role of contract formulation. Based on the assumption of uncertainty, the completeness of the transaction clauses signed by both parties is different from the completeness of the contingency-handling clauses. Based on the assumption of opportunistic behavior, when the other party violates the terms of the contract and carries out opportunistic behavior to its side, its side will choose different response strategies.

### Psychological Contract Theory

Psychological contract theory originated from organizational behavior and was initially applied in human resources, but now psychological contract theory is widely used in marketing research. Argyris and Ditz (1960) first proposed the concept of the psychological contract in his research but did not define it in detail. Levinson, the originator of the concept of the psychological contract, defines a psychological contract as an “unwritten contract” formed between an organization and its employees; in other words, the belief that an individual exchanges terms and conditions of a contract with another party (Robinson, 1996). In a marketing environment, a psychological contract is consumers' cognition and belief of implicit and unwritten mutual responsibility and obligation between themselves and enterprises (She et al., 2020). In the organizational environment, a psychological contract is a mutual agreement that restricts both employees and employers (Robbins, 2012). Based on the psychological contract perspective, Lusch and Brown (1996) studied the relationship between enterprises and channel dealers in channel transactions. The psychological contract can be divided into transactional and relational contracts, representing different orientations. Relational contracts mainly consider long-term interests, while transactional contract pays more attention to immediate interests. In the channel transaction, more attention is paid to the relationship contract, the contract between the two sides of the channel organization, organization and personnel, personnel and personnel. The contract will influence the behavior of the two sides of the channel members. Different types of psychological contract perception also affect opportunistic behaviors in channels (Chen et al., 2017). Based on this, this article argues that under the condition of cooperation between the two parties, when channel boundary personnel tolerate the opportunistic behavior of the other party based on work and personal tolerance, the perception of a psychological contract will affect the opportunistic behavior of border personnel.

### Omission Bias Theory

The omission bias originates from omission in jurisprudence, which refers to the legal actor who has a duty and should have the right and obligation to carry out a specific act but does nothing in the actual situation. There are usually two forms of omission, namely, to do nothing and to deliberately choose not to take action or not to change (Yeung et al., 2021). Inaction tendencies are linked to the “principle of harmful behavior,” which describes a phenomenon in which harm caused by behavior is often judged morally worse than harmful inaction (Hayashi and Mizuta, 2022). Therefore, when faced with the dilemma of action and inaction, both lead to similar adverse outcomes, people tend to choose omission (Yeung et al., 2021). Therefore, channel boundary personnel are more receptive to the opportunistic behavior caused by the other party's failure to fulfill its obligations or responsibilities and cannot tolerate the opportunistic behavior caused by the other party's failure to do what it can do (Seggie et al., 2013; Chen, 2017). The theory of omission bias is widely used in various fields, moral or legal judgments of various criminal acts due to omission; medical decisions on whether to vaccinate; and the harm of government personnel's inaction to citizens. There is still much room to explore the theory of omission bias in the study of marketing channels. Based on this theory, channel members can understand each other's attitude to opportunistic behavior in the transaction and choose different response strategies.

### Model Construction and Theoretical Hypothesis

**Figure 1:**
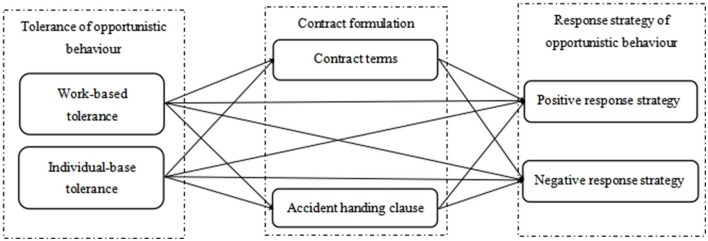
The relationship model between tolerance of opportunistic behavior, contract formulation, and response strategy of opportunistic behavior.

Based on other scholars' research, the theoretical model of this study is constructed by taking the tolerance of channel boundary personnel to opportunistic behaviors as antecedent variables, opportunistic behavior-coping strategies as outcome variables, and contract formulation as mediator variables, as shown in Figure 1.

The first is the relationship between opportunistic tolerance of channel boundary personnel and opportunistic behavior response strategies.

In channel trading, channel members will take opportunistic actions to maximize their interests (Williamson, 1975). First, as a member in the organization, the personnel of channel boundary may assume corresponding responsibilities, considering both sides' cooperation relationship in the organization, in the face of the other side of the opportunism behavior, will think the other party members have no respect for their own, destroyed the contract relationship, and are more reluctant to choose positive opportunism response strategy (passive acceptance and positive suggestions). Second, when the other party carries out opportunistic behavior, channel boundary personnel will think that the other party members do not act and tend to choose negative opportunistic behavior response strategies (expressing complaints, alienating relationships, threatening withdrawal and direct withdrawal) in order to ensure the maximization of organizational interests. Finally, with the increase in investment in proprietary assets in the transaction process, the barriers for both parties to withdraw from the relationship are higher (Williamson, 1985). They will not easily withdraw from the opportunistic behavior of the other party. Based on the above research, the following hypotheses are proposed:

H1a: The more the channel boundary personnel tolerate the other party's opportunistic behavior based on their work, the less they will choose the positive opportunistic behavior response strategy.

H1b: The more the channel boundary personnel tolerate the other party's opportunistic behavior based on their work, the more they will choose the negative opportunistic behavior response strategy.

Compared with the work-based tolerance of opportunistic behavior, individual-based tolerance of opportunistic behavior pays more attention to personal interests and personal emotions. The relationship in China is mainly reflected in human relations. The interpersonal relationship between border personnel on both sides in channel transactions is significant. When the border personnel believe that the two sides have a “psychological connection” through communication or contact (Zhuang et al., 2008), they will tolerate opportunistic behavior based on personal relationships. In the face of the opportunistic behavior of the other member, they will think that the other member has ignored the personal friendship accumulated by both sides, and the less their member will adopt the positive opportunistic behavior response strategy (passive acceptance and positive suggestions). In addition, according to the theory of omission bias, people are more willing to accept the other members due to not as opportunistic behavior (Chen, 2017), for the other members of the intended opportunistic behavior, their members will take opportunism behaviors of the adverse reaction strategy (threatening withdrawal, expressing complaints, alienating relationship and direct withdrawal). Based on the above research, the following assumptions are put forward:

H2a: Based on the individual, the more tolerant the opportunistic behavior of the other party, the less likely the channel boundary personnel will choose the positive opportunistic behavior response strategy.

H2b: The more individuals tolerate opportunistic behavior, the more they choose negative opportunistic behavior response strategies.

The second is the relationship between opportunistic behavior tolerance of channel boundary personnel and contract formulation.

In channel transactions, the contract terms made by channel members and the other party are also different based on their work and personal tolerance to opportunistic behavior. The other party acting beyond the scope of the contract will be more opportunistic and more likely to destroy a stable exchange environment (Qian and Liao, 2021). When the channel boundary personnel are more tolerant of each other's opportunistic behavior based on their work, considering the relationship between both sides form a contract, pay attention to the long-term cooperation of the guidance, the more do not want to let the other side to implement opportunism behavior destroy the relationship between the two sides, in the process of contract making use of trade terms and more detailed and more comprehensive accident-handling terms. When the channel boundary personnel are more tolerant of the opportunistic behavior of the other party, it indicates that both parties have a specific “human basis” and do not want to damage the relationship between them. However, interdependent channel partners need to use institutionalized and legalized contract management channels (Qian and Liao, 2021). They tend to choose more comprehensive contingency settlement clauses and more detailed transaction clauses to reduce conflicts and encourage cooperation. Based on this, the following hypotheses are proposed:

H3a: Channel boundary personnel's tolerance of opportunistic behavior based on work positively impacts the detail of transaction terms.

H3b: Channel boundary personnel's tolerance of opportunistic behavior based on work positively impacts the comprehensiveness of accident-handling clauses.

H3c: Channel boundary personnel's tolerance of opportunistic behavior positively affects the detail of transaction terms.

H3d: Channel boundary personnel's tolerance of opportunistic behavior positively affects the comprehensiveness of accident-handling clauses.

The third is the relationship between contract formulation and the choice of opportunistic response strategies.

The contract represents a formal relationship constraint with legal force, a written norm formed through formal negotiation (Zhou and Poppo, 2010). The contract terms include accident-handling terms and transaction terms. Transaction terms include generally predictable matters. Contingency clauses include future uncertainties in the contract terms (Luo, 2002). According to the neglect bias theory, one's side will perceive that the other side's tolerance of opportunistic behavior, whether based on work or personal, does not respect the agreement reached by both sides and is a manifestation of inaction in the transaction relationship. Therefore, the more detailed the transaction terms signed by both parties or the more comprehensive the contingency-handling terms will be, prompting channel boundary personnel to adopt negative opportunistic behavior response strategies (express complaints to stop loss in time, threaten to quit, alienate the relationship to ensure the protection of vested interests, withdraw to end the cooperative relationship with the other party). The more detailed the transaction terms signed by both parties or the more comprehensive the contingency-handling terms are, the more the channel boundary personnel are restrained from adopting positive opportunistic behavior response strategies (positive suggestions and passive acceptance are selected in turn). Based on the above discussion, this study proposes the following assumptions:

H4a: The details of transaction terms negatively affect the choice of a positive opportunistic behavior response strategy.

H4b: The comprehensiveness of the accident-handling clause hurts the choice of a positive opportunistic behavior response strategy.

H4c: The details of transaction terms positively affect their choice of a negative opportunistic behavior response strategy.

H4d: The accident-handling clause comprehensiveness positively impacts the choice of a negative opportunistic behavior response strategy.

The fourth is the mediating role of contract formulation.

Transaction cost theory believes that transaction uncertainty, that is, the probability of various risks occurring in the transaction process, is an important factor affecting transaction costs (Williamson, 1985). The contract stipulates the responsibilities and obligations of both parties, increasing the transparency of cooperation between the two parties (Tang et al., 2021) and reducing uncertainty (Kashyap and Murtha, 2017). Therefore, both companies will consider transaction costs and relationship coordination, jointly draft contract terms, and clear transaction items (Wang et al., 2019). The transaction clause in the contract can restrain each other's behavior and reduce conflicts. In case of uncertainty, the loss of both parties can be reduced, and the cooperative relationship can be maintained according to the contingency clause (Zhou et al., 2013). Channel border personnel, whether based on their job responsibilities within the organization, or based on the personal relationship status of opportunistic behavior to adopt a tolerant attitude, are considering there are certain dependencies on both sides, right now are more willing to use the prior consensus of formal contract to express emphasis on relations of cooperation, to guide subsequent transactions (Qian and Liao, 2021). Therefore, the opportunistic behavior tolerance of channel boundary personnel influences the choice of response strategy through contract formulation. Based on the above discussion, this study puts forward the following assumptions:

H5a: Channel boundary personnel choose positive opportunistic behavior response strategies based on job tolerance of opportunistic behavior through accident-handlingclauses.

H5b: Channel boundary personnel choose positive opportunistic behavior response strategies through transaction terms based on job tolerance of opportunism behavior.

H5c: Channel boundary personnel choose negative opportunistic behavior response strategies through accident-handling clauses based on job tolerance of opportunism behavior.

H5d: Channel boundary personnel choose negative opportunistic behavior response strategies through transaction terms based on job tolerance of opportunism behavior.

H5e: Based on the individual's tolerance of opportunistic behavior, channel boundary personnel can influence their choice of positive opportunistic behavior response strategy through accident-handling clause.

H5f: Based on the individual's tolerance of opportunistic behavior, channel boundary personnel influence their choice of positive opportunistic behavior response strategy through transaction terms.

H5g: Channel boundary personnel choose negative opportunistic behavior response strategies based on individual tolerance of opportunistic behavior through accident-handling clause.

H5h: Based on individual tolerance of opportunistic behavior, channel boundary personnel influence their response strategies to negative opportunistic behavior through transaction terms.

### Questionnaire Design and Research Samples

**Figure 2:**
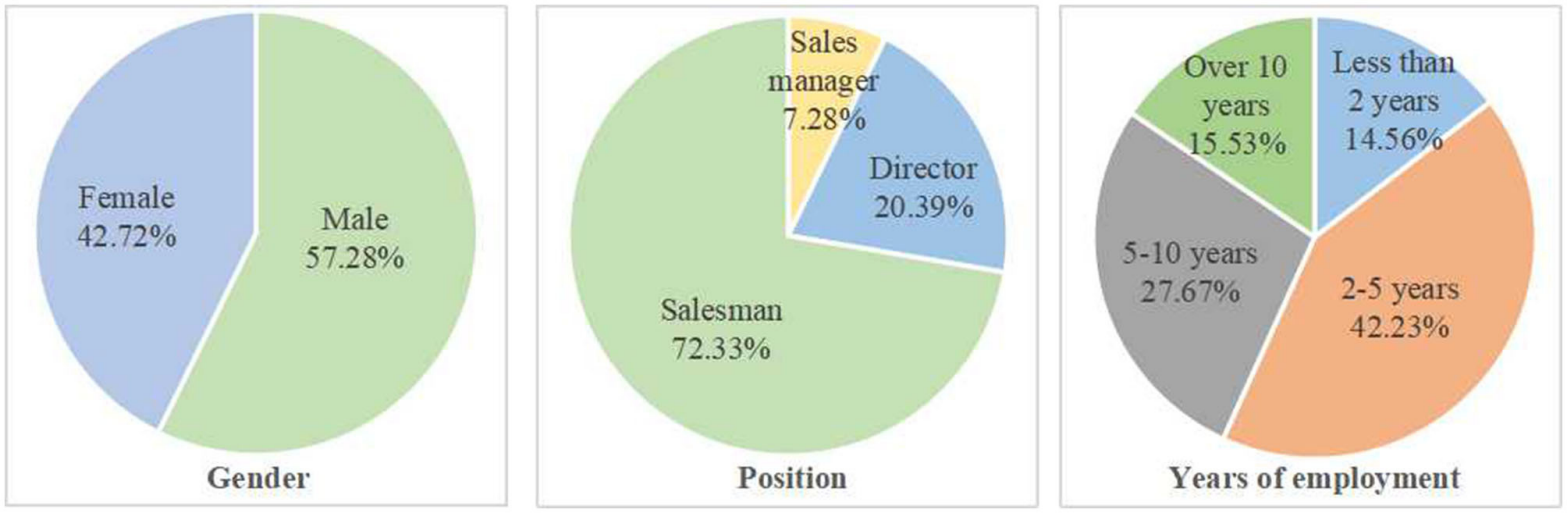
Sample statistical distribution results.

In the prediction stage, this study selects electronics industry, through electronic mail questionnaire, a total of 120 questionnaires, to take practical questionnaire analysis by SPSS software series, minor repairs on a questionnaire, delete the item title, not modify inaccurate expression language, and eventually formed the formal questionnaire for this study. During the formal investigation stage, select Beijing Zhongguancun electronic products channel boundary personnel as the research object, a total of 250 questionnaires were distributed, 220 copies were recovered, of which 206 valid questionnaires, the effective recovery rate was 82.4%, as shown in Figure 2.

The scales in this study are all from the mature scale. In order to make the items closer to the situation of the channel boundary personnel, the items are slightly modified in combination with the social background of Chinese human relations. All the measurement items of scale variables were measured by the Likert 7-point scoring method, with the degree from 1 to 7 becoming deeper and deeper, from completely disagree to completely agree. Respondents chose items from 1 to 7 according to their actual situation.

The opportunistic behavior tolerance scale refers to the scale developed by Chen (2017) and is modified in combination with the actual situation between social background and channels. Finally, it was determined that there are four items for individual-based opportunistic tolerance (TP) and four items for work-based opportunistic tolerance (TW). Opportunistic behavior reaction strategy references Zhang et al. (2016) and Zhang and Yin (2019) revised scale based on Ping (1993) division of opportunistic behavior reaction strategy and connecting with the social background and the actual situation of modified between channels, ultimately determining five items for passive acceptance (AC), four items for positive suggestions (VO), three items for expressing complaints (CO), five items for alienating relationships (NEG), five items for threatening exit (WIT), and three items for sign out (EX). The contract formulation measurement refers to the scale revised by Zhou et al. (2013) based on Lusch's contract division. It was modified according to the actual situation between the social background and the channel. Finally, it determined three items of the contract terms (EXC) and three items of the accident-handling clause (UEXP), as shown in Table 1.

**Table 1:** Questionnaire items and reliability test.

**Latent variable**	**Observation items**	**Factor load**	**Mean**	**SD**
Individual-based tolerance of opportunistic behavior AVE = 0.784, Cronbach's α = 0.908, CR = 0.927	TP1: Because of the personal relationship, to protect our interests, partners occasionally give us some untrue information, and we can often accept it.	0.913	4.859	1.5879
	TP2: Because of the personal relationship, sometimes the partner made promises to us on certain things, but then they did not really do it, and we often did not care about it.	0.863	4.835	1.5623
	TP3: Because of the personal relationship, sometimes the partner does not comply with the agreement reached with us to protect their interests, and we often do not hold it accountable.	0.871	4.854	1.6844
	TP4: Because of the personal relationship, sometimes partners have to hide some facts to get what they want from us, and we often tolerate it.	0.893	4.879	1.6586
Work-based tolerance of opportunistic behavior AVE = 0.770, Cronbach's α = 0.900, CR = 0.931	TW1: Based on the working relationship, we will tolerate the occasional behavior of partners who provide us with untrue information.	0.890	4.927	1.6078
	TW2: Based on the working relationship, we often tolerate the partner's failure to fulfill promises.	0.863	4.879	1.7022
	TW3: Based on the working relationship, we will tolerate the occasional non-compliance of the agreement with the manufacturer by the partner.	0.871	4.840	1.6075
	TW4: Based on the working relationship, we will tolerate the occasional behavior of the partner to conceal some facts.	0.886	4.913	1.5810
Passive acceptance AVE = 0.764, Cronbach's α = 0.921, CR = 0.942	AC1: If the partner has problems, I do not have to say anything to the partner because these problems will be solved by myself.	0.886	3.131	1.5640
	AC2: If there is a problem with this partner, I will ignore it because they will be resolved by themselves.	0.883	3.121	1.3968
	AC3: If there is a problem with the partner, the partner's problem and I will usually resolve it independently.	0.864	3.053	1.5994
	AC4: If there is a problem with this partner, I will ignore the problem with this supplier.	0.844	3.131	1.6579
	AC5: If there is a problem with this partner, I usually ignore it because they will be resolved by themselves.	0.892	3.068	1.6514
Positive suggestions AVE = 0.795, Cronbach's α = 0.913, CR = 0.939	VO2: If there is a problem with the partner, I will work with him to improve the situation.	0.892	4.971	1.6847
	VO3: If we face common problems, I will work with my partners to solve the problems we face together.	0.880	4.985	1.5726
	VO4: If we face a common problem, I will discuss any related issues with my partners.	0.884	4.888	1.6652
	VO5: If we face common problems, I will discuss our common problems with my partners.	0.909	4.791	1.6049
Express complaints AVE = 0.823, Cronbach's α = 0.926, CR = 0.949	CO1: If there is a problem with the partner, I will tell the partner that it is not feasible.	0.916	4.806	1.3868
	CO2: If there is a problem with the partner, I will tell the partner that his behavior is unacceptable.	0.891	4.684	1.5500
	CO3: If there is a problem with the partner, I will tell the partner I am dissatisfied.	0.901	5.010	1.6849
	CO4: If there is a problem with the partner, I will tell the partner that doing so will affect our cooperation.	0.921	5.010	1.6704
Estrangement AVE = 0.844, Cronbach's α = 0.906, CR = 0.942	NEG2: If we have a problem, I do not care about anything that happens as long as I get what I want.	0.940	3.170	1.7711
	NEG3: If we have a problem, I have given up paying attention to my partner and making the situation worse and worse.	0.910	3.248	1.5115
	NEG4: If we have a problem, I will look forward to the deterioration of the relationship with our partners.	0.906	3.223	1.6932
Threatening exit AVE = 0.804, Cronbach's α = 0.936, CR = 0.934	WIT1: I will tell the partner that I will consider ending the transaction relationship if the problem is not resolved.	0.902	3.087	1.6206
	WIT2: I will tell the partner that I may not continue maintaining the trading relationship with this partner if the problem is not resolved.	0.888	3.136	1.5177
	WIT3: I will tell the partner that I may consider changing to another partner if the problem is not resolved.	0.897	3.189	1.7216
	WIT4: I will tell the partner that I am looking for an alternative partner if the problem is not resolved.	0.896	3.209	1.3399
	WIT5: I will tell the partner that I will soon consider an alternative partner if the problem is not resolved.	0.900	2.913	1.8192
Sign out AVE = 0.810, Cronbach's α = 0.940, CR = 0.955	EX1: If there is a problem with this partner, I will end the relationship.	0.914	3.034	1.6181
	EX2: If there is a problem with this partner, I will not continue to maintain the relationship with this partner.	0.897	3.233	1.5219
	EX3: If there is a problem with this partner, I am looking for a new partner.	0.902	3.248	1.4456
	EX4: If there is a problem with the partner, I will change the partner at the right time.	0.885	3.209	1.7051
	EX5: If there is a problem with this partner, I terminate the trading relationship with this partner.	0.903	3.063	1.6589
Transaction clause AVE = 0.837, Cronbach's α = 0.896, CR = 0.939	EXC1: When dealing with our partners, our contract precisely defines the obligations of both parties.	0.914	4.791	1.6734
	EXC2: When dealing with our partners, we have a formal agreement detailing the responsibilities of both parties.	0.916	4.869	1.8232
	EXC3: When dealing with our partners, our contract stipulates how both parties implement the contract terms.	0.914	4.607	1.3813
Accident handling clause AVE = 0.784, Cronbach's α = 0.862, CR = 0.916	UEXP1: When dealing with our partners, our contract or distribution agreement stipulates legal remedies for failure to fulfill the contract terms.	0.896	4.675	1.5605
	UEXP2: When dealing with our partners, our contract or allocation agreement accurately explains what happens in unplanned events.	0.865	4.728	1.5090
	UEXP3: When dealing with our partners, our contract or distribution agreement explains exactly how to resolve the disagreement.	0.895	4.709	1.6087

### Statistical Methods

SPSS22.0 and AMOS20.0 software were used to analyze the collected data of 206 valid questionnaires. The items analyzed include (1) verifying the reliability and validity of the questionnaire, (2) the model fitting effect, (3) the standard method deviation analysis, and (4) the model path analysis and intermediary test.

## Data Analysis

### Reliability and Validity Test

SPSS22.0 software was used to test the reliability and validity of the scale. In the pre-investigation stage, the returned questionnaire was analyzed, and the items with factor loadings below 0.5 were deleted to form a formal measurement scale. Cronbach's α and CR of all latent variables are more significant than 0.8, indicating that the scale's reliability is good. The factor loadings are all >0.8, and the results show that the scale's validity in this study is good, as shown in Table 1. The Average Variance Extracted (AVEs) of all variables are more significant than 0.7. The square roots of the AVEs are all the more significant than the correlation coefficients, indicating that the discriminant validity of the model is good, as shown in Table 2.

**Table 2:** Discriminant validity test.

**Variable**	**TP**	**TW**	**AC**	**VO**	**CO**	**NEG**	**WIT**	**EX**	**EXC**	**UEXP**
TP	0.885									
TW	0.339^**^	0.877								
AC	−0.368^**^	−0.382^**^	0.874							
VO	0.413^**^	0.484^**^	−0.277^**^	0.892						
CO	0.410^**^	0.382^**^	−0.266^**^	0.245^**^	0.907					
NEG	−0.350^**^	−0.307^**^	0.231^**^	−0.268^**^	−0.229^**^	0.919				
WIT	−0.432^**^	−0.423^**^	0.301^**^	−0.294^**^	−0.333^**^	0.251^**^	0.901			
EX	−0.222^**^	−0.196^**^	0.122	−0.199^**^	−0.121	0.188^**^	0.170^*^	0.900		
EXC	0.323^**^	0.329^**^	−0.397^**^	0.252^**^	0.436^**^	−0.331^**^	−0.392^**^	−0.131	0.915	
UEXP	0.382^**^	0.365^**^	−0.256^**^	0.354^**^	0.363^**^	−0.305^**^	−0.314^**^	−0.200^**^	0.125	0.917

*The number on the diagonal represents the square root of AVE;*

** *p < 0.01,*

* *p < 0.05 (double tail)*.

### Evaluation of the Overall Model Fitting Effect

This study uses channel boundary personnel's tolerance to opportunistic behavior as the antecedent variable, opportunistic behavior response strategy as the outcome variable, and contract formulation as an intermediary variable to establish a structural equation model (SEM) to explore channel boundary personnel's tolerance behavior against opportunism research on the influence of behavioral response strategy choices and the mediating role of contract formulation. The 206 questionnaires returned were verified using the AMOS 20 pair model. Through the analysis of the model, root mean square error of approximation (RMSEA) = 0.023, Chi-square degree of freedom (CMIN/DF) = 1.111, goodness of fit index (GFI) = 0.922, normed fit index (NFI) = 0.926, increment fit index (IFI) = 0.992, and comparative fit index (CFI) = 0.992. All the above indexes are within the standard range, indicating that the model fitting effect is good, as shown in Table 3.

**Table 3:** Overall evaluation index of structural equation model.

**Statistical test volume**	**CMIN**	**CMIN/DF**	**RMSEA**	**GFI**	**NFI**	**RFI**	**IFI**	**TLI**	**CFI**	**PGFI**	**PNFI**	**PCFI**
Index value	174.405	1.111	0.023	0.922	0.926	0.911	0.992	0.990	0.992	0.689	0.765	0.820

### Model Path Analysis

SEM was used to analyze the hypothetical path, as shown in Table 4. First, channel boundary work-based tolerance of opportunistic behavior negatively affects the positive response strategies (ß = −0.53, *p* = 0.001). Work-based tolerance of opportunistic behavior positively impacts negative response strategies (ß = 0.29, *p* = 0.000); H1a and H1b were verified. At the same time, channel boundary personnel individual-based tolerance of opportunistic behavior has a significant negative impact on positive response strategies (ß = −0.39, *p* = 0.000) and a significant positive effect on negative response strategies (ß = 0.37, *p* = 0.000); H2a and H2b was verified.

**Table 4:** Results of path analysis.

**Hypothesis**	**Relationship**	**Path coefficient**	**C.R**.	***P*** **-value**	**Result**
H1a	TW → PRS	−0.53	−3.603	0.001*	Supported
H1b	TW → NRS	0.29	3.467	***	Supported
H2a	TP → PRS	−0.39	−3.603	***	Supported
H2b	TP → NRS	0.37	4.364	***	Supported
H3a	TW → CT	0.26	3.280	0.001*	Supported
H3b	TW → AHC	0.29	3.701	***	Supported
H3c	TP → CT	0.25	3.213	0.001*	Supported
H3d	TP → AHC	0.37	3.922	***	Supported
H4a	CT → PRS	−0.27	−2.710	0.007**	Supported
H4b	AHC → PRS	−0.53	−1.892	0.058	Not supported
H4c	CT → NRS	0.46	5.486	***	Supported
H4d	AHC → NRS	0.29	3.662	***	Supported

*** *p < 0.001,*

** *p < 0.01,*

* *p < 0.05*.

Second, as for the relationship between border personnel's tolerance of opportunistic behavior and contract formulation, the channel border personnel work-based tolerance of opportunistic behavior has a significant positive impact on the completeness of transaction clauses (ß = 0.26, *p* = 0.001) and a significant positive impact on the comprehensiveness of accident-handling clauses (ß = 0.29, *p* = 0.000); H3a and H3b were verified. At the same time, channel boundary personnel based on personal tolerance of opportunistic behavior have a significant positive impact on the completeness of transaction clauses (ß = 0.25, *p* = 0.001), and individual tolerance to opportunistic behavior has a significant positive impact on the comprehensiveness of contingency provisions (ß = 0.37, *p* = 0.000); thus, H3c and H3d were verified.

Finally, the path of contract formulation and response strategy is analyzed. The transaction clauses negatively influence boundary personnel to choose a positive opportunistic response strategy (ß = −0.27, *p* = 0.007) and cheerful influence border personnel to adopt negative opportunistic behavior response strategy (ß = 0.46, *p* = 0.000). Similarly, contingency clauses positively influence border personnel to adopt negative opportunistic behavior response strategies (ß = 0.29, *p* = 0.000). However, the impact path of contingency clause on positive opportunistic behavior is not significant (β = −0.53, *p* = 0.058 > 0.050). In summary, H4a, H4c, and H4d have been verified, while H4b has not.

### Analysis of Mediating Effect

This article uses the bootstrap confidence interval method to verify the mediating effect. The process plug-in of SPSS was used for intermediary analysis. For the four groups of mediating hypothesis, bootstrap ML, repeated sampling 5,000 times (>1,000), and using bias-corrected 95% confidence interval (LLCI, ULCI) to test the mediating effect, the analysis results are shown in Table 5. The confidence intervals of H5b, H5e, and H5f include 0 value. That is, the mediating effect cannot be verified; other mediating effects were verified (confidence intervals excluding 0 and significant).

**Table 5:** Intermediary inspection results.

**Hypothesis**	**Mediating effect**	**Coefficient**	**Boot SE**	**95% confidence interval of deviation correction**		***P*** **-value**	**Result**
				**Boot LLCI**	**Boot ULCI**		
H5a	TW → UEXP → POS	0.0554	0.0217	0.0126	0.0981	0.05*	Partial mediation
H5b	TW → EXC → POS	−0.0058	0.0194	−0.0440	0.0324	0.058	Not supported
H5c	TW → UEXP → NEG	0.0789	0.0322	0.0135	0.1442	***	Partial mediation
H5d	TW → EXC → NEG	0.1617	0.0789	0.1068	0.2166	***	Partial mediation
H5e	TP → UEXP → POS	0.0177	0.0218	−0.0253	0.0608	0.75	Not supported
H5f	TP → EXC → POS	0.0160	0.0197	−0.0229	0.0549	0.055	Not supported
H5g	TP → UEXP → NEG	0.1173	0.0321	0.0540	0.1806	***	Partial mediation
H5h	TP → EXC → NEG	0.1647	0.0281	0.1093	0.2201	***	Partial mediation

*** p < 0.001, ^**^p < 0.01,

* *p < 0.05*.

## Results

In this study, 206 valid questionnaires were collected and analyzed, combined with SEM and effect analysis. The empirical results verified H1a, H1b, H2a, H2b, H3a, H3b, H3c, H3d, H4c, and H4d. The H4b effect is not significant, which may be because the more comprehensive the contract contingency treatment clauses are when the other party carries out opportunistic behavior, channel boundary personnel tend to choose positive response strategies based on their personal belief that the other party member does not act. The mediating effects of H5a, H5c, H5d, H5g, and H5h were verified, but H5b, H5e, and H5f were not verified. The mediating effect of the contingency clause on the choice of response strategy based on job tolerance to opportunistic behavior and positive opportunistic behavior has not been verified. On the one hand, it may be due to the small number of samples, and the corresponding conclusions cannot be drawn. On the other hand, if H4b is not verified, H5e is not verified, as shown in Table 6.

**Table 6:** Summary of hypothesis validation.

**Item**	**Hypothesis**	**Result**
H1a	The more the channel boundary personnel tolerate the other party's opportunistic behavior based on their work, the less they will choose the positive opportunistic behavior response strategy.	Supported
H1b	The more the channel boundary personnel tolerate the other party's opportunistic behavior based on their work, the more they will choose the negative opportunistic behavior response strategy.	Supported
H2a	Based on the individual, the more tolerant the opportunistic behavior of the other party, the less likely the channel boundary personnel will choose the positive opportunistic behavior response strategy.	Supported
H2b	The more individuals tolerate opportunistic behavior, the more they choose negative opportunistic behavior response strategies.	Supported
H3a	Channel boundary personnel's tolerance of opportunistic behavior based on work positively impacts the detail of transaction terms.	Supported
H3b	Channel boundary personnel's tolerance of opportunistic behavior based on work positively impacts the comprehensiveness of accident handling clauses.	Supported
H3c	Channel boundary personnel's tolerance of opportunistic behavior positively affects the detail of transaction terms.	Supported
H3d	Channel boundary personnel's tolerance of opportunistic behavior positively affects the comprehensiveness of accident handling clauses.	Supported
H4a	The details of transaction terms negatively affect the choice of a positive opportunistic behavior response strategy.	Supported
H4b	The comprehensiveness of the accident handling clause hurts the choice of a positive opportunistic behavior response strategy.	Not supported
H4c	The details of transaction terms positively affect their choice of a negative opportunistic behavior response strategy.	Supported
H4d	The accident handling clause comprehensiveness positively impact the choice of a negative opportunistic behavior response strategy.	Supported
H5a	Channel boundary personnel choose positive opportunistic behavior response strategies based on job tolerance of opportunistic behavior through accident handling clauses.	Supported
H5b	Channel boundary personnel choose positive opportunistic behavior response strategies through transaction terms based on job tolerance of opportunism behavior.	Not supported
H5c	Channel boundary personnel choose negative opportunistic behavior response strategies through accident handling clauses based on job tolerance of opportunism behavior.	Partial mediation
H5d	Channel boundary personnel choose negative opportunistic behavior response strategies through transaction terms based on job tolerance of opportunism behavior.	Partial mediation
H5e	Based on the individual's tolerance of opportunistic behavior, channel boundary personnel can influence their choice of positive opportunistic behavior response strategy through accident handling clause.	Not supported
H5f	Based on the individual's tolerance of opportunistic behavior, channel boundary personnel influence their choice of positive opportunistic behavior response strategy through transaction terms.	Not supported
H5g	Channel boundary personnel choose negative opportunistic behavior response strategies based on individual tolerance of opportunistic behavior through accident handling clause.	Partial mediation
H5h	Based on individual tolerance of opportunistic behavior, channel boundary personnel influence their response strategies to negative opportunistic behavior through transaction terms.	Partial mediation

## Discussion

### Implications

First, channel transaction is a process of the repeated game. Members of both sides should strengthen communication with each other, understand each other's acting style and worldly attitude, promote the establishment of trust mechanism of both sides, establish a good working relationship and deep personal affection, and achieve a long-term and stable cooperative relationship. Second, the members of both sides of the channel should respect the established cooperative relationship in the transaction process and not attempt to increase their interests through opportunistic behavior at the expense of the partner's interests, which will outweigh the gain. Finally, if both parties want to cooperate for a long time, it is necessary to formulate a contract to constrain the behaviors of both parties. The more detailed the terms of the transaction, the clearer the responsibilities and obligations of both parties. The more comprehensive the contingency clause, the better it will consider all circumstances.

## Conclusion

Based on transaction cost theory, psychological contract theory, and neglect bias theory, this study constructed a theoretical model with channel boundary personnel's tolerance to opportunism as the independent variable, opportunistic behavioral response strategy choice as the dependent variable, and contract formulation as the intermediary variable. SPSS and AMOS were used to analyze the data of 206 valid questionnaires, and the conclusions were as follows.

First, both parties establish a specific basis of trust and reach a psychological contract in marketing channel transactions. If one channel member is more tolerant of the opportunistic behavior of the other side, he or she is more inclined to choose a negative response strategy, and the more intolerant he or she is, he or she will choose a positive response strategy. When formulating the contract, channel boundary personnel, from the perspective of work, will use more detailed transaction terms and more comprehensive contingency-handling terms to restrain the other party from reducing the frequency and probability of the opportunistic behavior of the other party.

Second, in the repeated game between the two sides of the channel, the emotional basis based on individuals has been established. If one channel member is more tolerant of the other party's opportunistic behavior based on a personal relationship, it will choose the negative response strategy. For the sake of long-term interests, various factors will be considered, and detailed general items will be formulated to constrain the behaviors of both parties and reduce the losses of both parties. At the same time, members of both sides will sign more comprehensive accident-handling clauses. When accidents occur in cooperation, both sides can deal with them according to the contract to avoid friction between the two sides.

Third, transaction terms positively affect the choice of negative response strategies based on work and individual opportunistic behavior tolerance and have no intermediary effect on positive response strategies. The more complex the contract terms, the other party's opportunistic behavior will be disloyal to the relationship between the two parties. Whether based on their work or personal tolerance to the opportunistic behavior of the other party, their personnel are more inclined to choose negative response strategies. The contingency clause has no mediating effect on positive response strategies based on individual opportunistic behavior tolerance. The more comprehensive the contingency clause is, the space for both sides of the channel to implement opportunistic behavior will be reduced. Once the other side implements opportunistic behavior, the border personnel of their side will perceive the inaction of the channel members of the other side no matter from the perspective of work or personal and are more inclined to choose negative response strategies.

### Limitations and Future Directions

This article has the following research limitations. First, research externalities cannot be guaranteed. The data collected in this study are from only one party. After a comprehensive analysis, future research on channel behavior should collect data from both parties to draw more accurate conclusions. At the same time, this study only collects data from the electronics industry. Data analysis of multiple industries should be collected as much as possible to draw more general conclusions in future studies. Second, the selection of control variables is insufficient. The control variables in this study are mainly the company's size, years of work, gender, age, and other aspects. In future studies, the selection of control variables should be based on factors to make the research results accurate.

## Data Availability Statement

The raw data supporting the conclusions of this article will be made available by the authors, without undue reservation.

## Ethics Statement

Ethical review and approval was not required for the study on human participants in accordance with the local legislation and institutional requirements. Written informed consent for participation was not required for this study in accordance with the national legislation and the institutional requirements.

## Author Contributions

JC made a theoretical construction and completed the summary of the article. RH was responsible for collecting and analyzing data. ZL made translation and article reviews. All authors contributed to this article and approved the version submitted.

## Funding

This research was supported by the National Natural Science Foundation of China (71562003).

## Conflict of Interest

The authors declare that the research was conducted in the absence of any commercial or financial relationships that could be construed as a potential conflict of interest.

## Publisher's Note

All claims expressed in this article are solely those of the authors and do not necessarily represent those of their affiliated organizations, or those of the publisher, the editors and the reviewers. Any product that may be evaluated in this article, or claim that may be made by its manufacturer, is not guaranteed or endorsed by the publisher.
